# Choroid Plexus Volume, Amyloid Burden, and Cognition in the Alzheimer’s Disease Continuum

**DOI:** 10.14336/AD.2024.0118

**Published:** 2024-01-18

**Authors:** Seong Ho Jeong, Chae Jung Park, Jungho Cha, Sang-Young Kim, Seung-Koo Lee, Yun Joong Kim, Young H. Sohn, Seok Jong Chung, Phil Hyu Lee

**Affiliations:** ^1^Department of Neurology, Inje University Sanggye Paik Hospital, Seoul, Korea.; ^2^Department of Neurology, Yonsei University College of Medicine, Seoul, Korea.; ^3^Department of Radiology, Yongin Severance Hospital, Yonsei University Health System, Yongin, Korea.; ^4^Nash Family Center for Advanced Circuit Therapeutics, Icahn School of Medicine at Mount Sinai, New York, NY, USA.; ^5^MR Clinical Science, Health Systems, Philips Korea, Seoul, Korea.; ^6^Department of Radiology, Severance Hospital, Research Institute of Radiological Science and Centre for Clinical Imaging Data Science, Yonsei University College of Medicine, Seoul, Korea.; ^7^Department of Neurology, Yongin Severance Hospital, Yonsei University Health System, Yongin, Korea.; ^8^YONSEI BEYOND LAB, Yongin, Korea.

**Keywords:** Alzheimer’s disease;, choroid plexus, amyloid burden, cognition

## Abstract

As a part of the glymphatic system, the choroid plexus (CP) is involved in the clearance of harmful metabolites from the brain. We investigated the association between CP volume (CPV), amyloid-β (Aβ) burden, and cognition in patients on the Alzheimer’s disease (AD) continuum. We retrospectively reviewed the records of 203 patients on the AD continuum and 82 healthy controls who underwent brain magnetic resonance imaging and ^18^F-florbetaben positron emission tomography. Automatic segmentation was performed, and the CPV was calculated. Cognitive function was assessed using detailed neuropsychological tests, and patients on the AD continuum were categorized into the non-dementia and dementia groups. The relationships between CPV, Aβ burden, and cognitive function were assessed using multivariate linear regression and linear mixed model. CPV was greater in the AD group than in the healthy control group (1.50 vs. 1.30, *P* < 0.001), but was comparable between the AD non-dementia and dementia groups (1.50 vs. 1.48, *P* = 0.585). After adjusting for age and sex, a larger CPV was significantly associated with greater global Aβ deposition (β = 0.20, P = 0.002). Larger CPV was also associated with worse general cognitive function assessed using the sum of boxes of the clinical dementia rating scale (*β* = 0.85, *P* = 0.034) and lower composite scores for memory (β = -0.68, P = 0.002) and frontal/executive function domains (β = -0.65, P < 0.001). In addition, a larger CPV was associated with a more rapid decline in Mini-Mental State Examination scores in the AD dementia group (*β* = -0.58, *P* = 0.004). The present study demonstrated that CP enlargement was associated with increased Aβ deposition and impaired memory and frontal/executive function in patients on the AD continuum.

## INTRODUCTION

Alzheimer's disease (AD) is characterized by the presence of extracellular plaques containing amyloid-β (Aβ) aggregates and intracellular tangles containing aggregated tau proteins in the brain [1]. Recent research has indicated that the inability to remove these proteins, rather than their overproduction, is responsible for AD development [2, 3]. Although earlier clinical trials of monoclonal antibodies for Aβ in AD failed to demonstrate their efficacy [4, 5], recent clinical studies have continuously shown that monoclonal antibodies for Aβ eradicate brain Aβ retention and have beneficial effects on clinical outcome [6, 7], which supports the aberrant accumulation of Aβ underlying the development of AD. Several clearance systems and mechanisms are involved in the removal of these proteins, including transport across the blood-brain and blood-cerebrospinal fluid (CSF) barriers, interstitial fluid bulk flow, and CSF absorption into the circulatory and glymphatic systems [3].

The choroid plexus (CP) is a highly vascularized structure found in the ventricles which is responsible for CSF production. Its function in neurodegenerative disorders has become a topic of interest, owing to its association with the glymphatic drainage system and neuroinflammation. Several preclinical studies have shown that the CP plays an important role in the removal of Aβ [8, 9]. Additionally, autopsy studies have indicated that morphological changes in the CP can be observed in AD populations [10-12], providing evidence for a role of CP dysfunction in AD. A recent *in vivo* study demonstrated that the CP volume (CPV) is larger in patients with AD in more severe stages of the disease [13]. Another study demonstrated that CPV is negatively associated with CSF protein levels (total-tau and phosphorylated-tau) in patients with AD [14], indicating a possible role of the CP in the clearance of CSF proteins. However, a recent study failed to demonstrate an association between CPV and Aβ positivity in AD [13], although the study had some limitations in that only qualitative analyses (Aβ positivity on visual rating) were performed on a limited number of the enrolled participants (94 of 532 patients). Also, more than 30% of the mild cognitive impairment (MCI) group and 20% of the AD group showed no evidence of Aβ deposition on positron emission tomography (PET) scans, suggesting that a substantial proportion of participants had non-AD pathology. The exact relationship between CP dysfunction and misfolded proteins in AD, therefore, remains unclear.

Therefore, considering that CP is associated with glymphatic function and the clearance of misfolded protein, we hypothesized that CPV is associated with Aβ retention in patients on the AD continuum. In the present study, we investigated the association between CPV calculated from 3D T1-weighted MRI and Aβ deposition estimated from 18F-florbetaben (18F-FBB) PET. Furthermore, we investigated whether CPV was related to cognitive decline.

## MATERIALS AND METHODS

### Participant characteristics

We retrospectively reviewed the medical records of 208 consecutive patients on the AD continuum from the Severance Hospital who visited our clinic due to subjective symptoms of cognitive impairment between June 2015 and May 2020. These patients underwent brain magnetic resonance imaging (MRI) and exhibited Aβ positivity on ^18^F-FBB PET scans. All participants had been previously investigated in our previous study [15]. A detailed neuropsychological study was performed to assess the level of cognitive impairment in patients with cognitive complaints. Patients with no evidence of cognitive impairment and MCI in the detailed neuropsychological study were categorized into “preclinical AD” and “prodromal AD” according to the NIA-AA guidelines, respectively [16, 17]. Patients with dementia were categorized into the AD dementia group [18]. Since patients with preclinical AD (n = 14) accounted for less than 10% of all study participants, we combined the preclinical and prodromal AD groups into the AD non-dementia group. None of the participants in the control group (n = 82) complained of cognitive impairment, and all exhibited normal cognitive function according to the Korean version of the Mini-Mental State Examination (MMSE) and detailed neuropsychological tests. This study was approved by the Institutional Review Board of the Yonsei University Severance Hospital. The need for informed consent was waived for patients on the AD continuum (IRB No. 4-2021-0759) because of the retrospective nature of the study. Informed consent for participation was obtained from all healthy controls (IRB No. 4-2015-0551).

### Imaging procedures of ^18^F-FBB and MRI scans

^18^F-FBB PET scans were obtained using a Discovery 600 system (General Electric Healthcare, Milwaukee, MI, USA). ^18^F-FBB-PET images were acquired 90 min after the administration of 300MBq (8mCi) FBB for 20 min. Images were acquired with a 256 × 256 matrix and reconstructed with an ordered-subsets expectation maximization algorithm in an iso-0.98-mm voxel size. Acquisition corrections included decay, attenuation, scatter, dead time, normalization, sensitivity calibration using a dose calibrator, and random correction.

All patients underwent brain MRI using a 3.0T scanner (Achieva; Philips Medical Systems, Best, Netherlands) with a 32-channel receiver array head coil. The head motion was minimized by restraining the foam pads provided by the manufacturer. A high-resolution, T1-weighted MRI volume data set was obtained from all subjects with a 3-dimensional T1-TFE sequence configured with the following acquisition parameters: axial acquisition with a 224 × 256 matrix; 256 × 256 reconstructed matrix with 182 slices; 220-mm field of view; 0.98 × 0.98 × 1.2 mm^3^ voxels; 4.6 ms echo time; 9.6 ms repetition time; 8° flip angle; and 0 mm slice gap.

### Visual assessment of ^18^F-FBB scans

Brain amyloid plaque load (BAPL) scores [19] were assessed based on visual ratings by an expert reader who was blinded to the clinical diagnosis. A BAPL score of 1 was classified as Aβ-negative, whereas scores of 2 and 3 were classified as Aβ-positive, and all participants enrolled in this study had BAPL scores of 2 or 3. CT scans were performed along with ^18^F-FBB PET scans to improve the accuracy of the interpretation. Aβ load in the occipital lobe was not considered when deciding Aβ positivity.

### Quantitative analyses of ^18^F-FBB scans

All image processing was performed using the Statistical Parametric Mapping 12 (SPM12; Wellcome Trust Centre for Neuroimaging, London, UK; www.fil.ion.ucl.ac.uk/spm/) and FreeSurfer 6.0 (https://surfer.nmr.mgh.harvard.edu/) software. The processing was performed in a manner similar to that previously described for the ADNI PET pipeline at Berkeley [20]. The PET images were co-registered onto the corresponding structural MRI scans. The structural MR scans of each patient were segmented using FreeSurfer to define reference and target cortical regions. The SUVRs in the five FreeSurfer-defined cortical target regions, including the frontal (all cortices anterior to the precentral sulcus), parietal (supramarginal gyrus, inferior/superior parietal lobules, and precuneus), lateral temporal (middle and superior temporal gyri), and anterior/posterior cingulate, were calculated by dividing the uptake in these regions by that in the whole-cerebellum. Global SUVRs were calculated as the volume-weighted mean across the four cortical regions (frontal, parietal, lateral temporal, and anterior/posterior cingulate cortices) [21].

### MRI analyses

**Figure 1.**
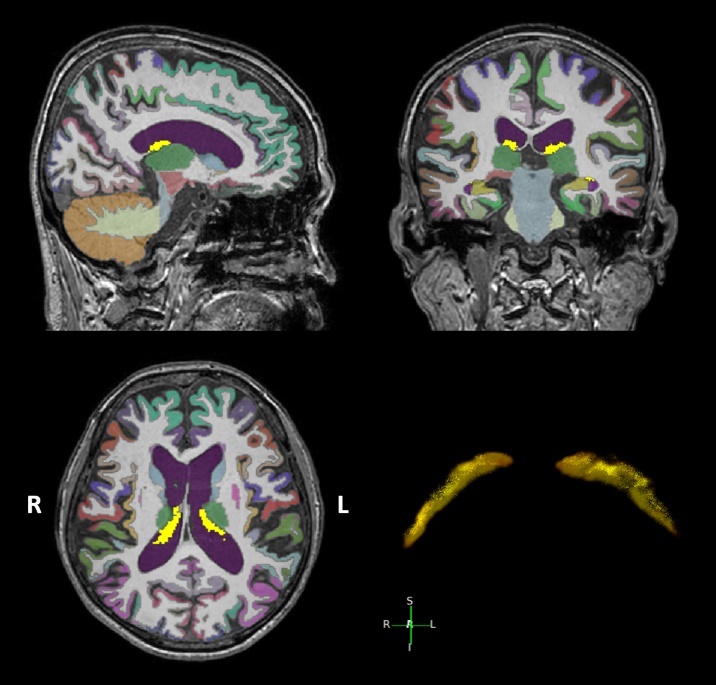
**A representative automatic segmentation results from a patient using FastSurfer**. The CP volume labels are highlighted in yellow. For visualization purposes, a 3D rendered CP mask is shown at the bottom right. CP = choroid plexus

Of the 208 patients with AD, 5 were excluded from this investigation because of preprocessing errors in the T1-weighted images. Therefore, 203 patients with AD and 82 controls were included in the MRI analysis. Automated segmentation of brain regions and the cortical thickness values of each region were extracted using FastSurfer, which is a fast and extensively validated deep-learning pipeline for the fully automated processing of structural human brain MRIs [22]. This software enabled whole-brain segmentation into 95 classes within 50 seconds per subject. The details of this program and its validity have been shown elsewhere (https://deep-mi.org/research/fastsurfer/). A representative automatic segmentation of the CP using FastSurfer is shown in Figure 1. In a subset of 30 patients, we manually segmented the CPV to assess inter-method reliability. One neuroradiologist (C.J.P., with five years of experience) manually segmented the CP in the lateral ventricle in the 3D T1-weighted volumetric images using 3D Slicer version 5.0.3 (www.slicer.org). From these images, the intracranial volume (ICV), lateral ventricle volume (LVV), white matter hyperintensity volume (WMHV), and hippocampal volume were all measured. All final automatic segmentation results were checked and approved by a single neuroradiologist (C.J.P.) without subsequent manual correction. The regional volumes were expressed as the ratio of the regional volume to the total ICV (ratio of ICV × 10^3^).

To measure the accuracy of automatic segmentation, we calculated the Dice similarity coefficients (DSC) between the manually and automatically segmented regions of interest (ROIs) of the CPs. The Dice score on tumor areas is defined as follows [23]:

$$\mathrm{DSC}=\frac{2\times \left|S\cap M\right|}{\left|S\right|+\left|M\right|}$$

where S and M represent an automatic segmentation and the corresponding manual segmentation, respectively. A Dice score of 1.0 corresponds to perfect overlap, while a score of 0.0 indicates no overlap.

### Neuropsychological tests

At the initial assessment, 199 (98.0%) patients on the AD continuum and all participants in the normal control group underwent a standardized neuropsychological battery test called the Seoul Neuropsychological Screening Battery [24, 25], which comprises tests that assess attention/ working memory, language, visuospatial, memory, and frontal/executive function. Standardized *z*-scores were available for all scorable tests, based on the age- and education-matched norms. Participants performed the following tests: two tests (digit span forward and backward) for the attention/working memory function domain; one test (the Korean version of the Boston Naming Test) for the language function domain; one test (copying item of the Rey-Osterrieth Complex Figure Test [RCFT]) for the visuospatial function domain; six tests (immediate recall, 20 min delayed recall, and recognition items of the Seoul Verbal Learning Test and the RCFT) for the memory function domain; and three tests (the Category Fluency Test [animals and groceries] for semantic fluency, the Controlled Oral Word Association Test for phonemic fluency, and the Stroop color reading test) for the frontal/executive function domain. A composite score was calculated for each cognitive domain by dividing the sum of the *z*-scores of individual tests by the number of tests. The operational definition of cognitive impairment that we used has been described in a previous study [26]. In addition, the Korean version of the MMSE and Clinical Dementia Rating-Sum of Boxes (CDR-SOB) were applied to assess the participants’ general cognition [27]. Cognitive status was established through a consensus among two neurologists and one neuropsychologist based on evidence of abnormal activities of daily living (ADL), assessed both clinically and based on instrumental ADL scales [28, 29].

### Longitudinal assessment of the changes in MMSE score over time

Of the 203 patients with AD continuum, 56 were excluded from the longitudinal analysis for cognitive decline due to the following reasons: (1) unavailability of longitudinal MMSE data in 54 patients and (2) enrollment of 2 patients in a clinical trial of anti-amyloid therapy. Thus, 147 patients were included in the longitudinal analysis. The mean follow-up duration was 2.0 ± 0.9 years.

### Statistical analysis

The baseline clinical, neuropsychological, and imaging characteristics of the study participants were analyzed using analysis of variance for continuous variables, whereas the chi-square or Fisher’s exact tests were used to analyze categorical variables. Correlation analyses were performed to identify the relationship between CPV and other imaging parameters. Normality of distribution was assessed using Shapiro-Wilk test.

Correlation analyses were performed for all patients on the AD continuum, as well as healthy controls, to investigate the relationship between CPV and global or regional SUVRs on ^18^F-FBB PET scans. Multivariate linear regression analysis for global or regional SUVRs was performed to explore the independent effect of CPV on the baseline Aβ burden after adjusting for age and sex, which are known factors related to Aβ deposition [30, 31]. The false discovery rate (FDR) method was used to correct multiple tests for the four regional SUVRs.

In terms of the association between CPV and cognitive function, multivariable linear regression analysis was used to determine the independent effects of CPV on the composite scores of each cognitive function domain, while adjusting for the effects of sex and cognitive status in all participants, including healthy controls and patients on the AD continuum. Age and years of education were not used as covariates because the composite score of each cognitive function domain was calculated using *z*-scores based on age- and education-matched norms. The FDR method was used to correct multiple tests for the five cognitive function domains. When determining the effect of CPV on CDR-SOB, we additionally adjusted for age at the time of MRI and years of education.

As described above, in this study, we included healthy controls and patients on the AD continuum in the correlation and multivariable linear regression analyses in order to investigate the brain and cognitive changes encompassing the transition from healthy aging to prodromal AD and AD dementia, without restricting the analysis solely to disease progression. As a sensitivity analysis, we performed all the aforementioned analyses within the whole AD (non-dementia + dementia) group, AD non-dementia group, and AD dementia group, and the APOE ε4 carrier status was additionally adjusted for in the multivariable linear regression analyses.

A linear mixed model was used to assess the rate of longitudinal changes in the MMSE scores according to the CPV in 147 patients on the AD continuum after adjusting for age, sex, years of education, APOE ε4 carrier status, cognitive status, global Aβ SUVRs, CPV, time, and a CPV × time interaction term. The effect of CPV on longitudinal MMSE score change over time was tested using a CPV × time interaction term. Subgroup analyses according to the cognitive status (non-dementia and dementia subgroups) were performed by using the same statistical model.

Statistical analyses were performed using the R software (v4.0, http://www.r-project.org). Results with a two-tailed *P* value or FDR-corrected *P* value < 0.05 were considered statistically significant.

**Table 1:** Demographic and clinical characteristics of study participants.

	Normal control (n = 82)	AD non-dementia (n = 126)	AD dementia (n = 77)	*P*
**Demographic characteristics**				
**Age at MRI scan**	66.46 ± 9.36	74.94 ± 7.23	76.31 ± 6.74	<0.001^a^,^b^
**Female, n (%)**	44 (53.66%)	73 (57.94%)	53 (68.83%)	0.130
**Education, y**	15.34 ± 4.14	11.48 ± 4.96	9.52 ± 5.47	<0.001^a^,^b^,^c^
**MMSE**	29.00 ± 1.32	24.50 ± 3.10	20.65 ± 3.96	<0.001^a^,^b^,^c^
***APOE* ε4 carrier**		65 (51.59%)	41 (53.25%)	0.932
**Vascular risk factors**				
**Hypertension**	22 (26.8%)	64 (50.8%)	43 (55.8%)	<0.001^a^,^b^
**Diabetes mellitus**	8 (9.8%)	31 (24.6%)	13 (16.9%)	0.024^a^,^b^
**Dyslipidemia**	21 (25.6%)	37 (29.4%)	22 (28.6%)	0.835
**Neuropsychological tests**				
**Attention/working memory**	0.80 ± 0.80	-0.08 ± 0.90	-0.41 ± 0.79	<0.001^a^,^b^,^c^
**Language**	0.48 ± 0.67	-0.70 ± 1.56	-1.29 ± 1.44	<0.001^a^,^b^,^c^
**Visuospatial**	0.26 ± 0.62	-0.64 ± 2.02	-1.26 ± 2.56	<0.001^a^,^b^,^c^
**Memory**	0.48 ± 0.53	-1.13 ± 0.87	-1.54 ± 0.77	<0.001^a^,^b^,^c^
**Frontal/executive**	0.37 ± 0.59	-0.59 ± 0.71	-1.12 ± 0.84	<0.001^a^,^b^,^c^
**CDR-SOB**	0.06 ± 0.16	1.64 ± 1.05	4.22 ± 2.30	<0.001^a^,^b^,^c^
**Volumetric MRI measures**				
**CPV, ratio of ICV × 10^3^**	1.30 ± 0.26	1.50 ± 0.26	1.48 ± 0.26	<0.001^a^,^b^
**LVV, ratio of ICV × 10^3^**	17.82 ± 8.17	25.64 ± 9.72	28.99 ± 10.72	<0.001^a^,^b^
**Hippocampal volume, ratio of ICV × 10^3^**	5.88 ± 0.56	5.03 ± 0.66	4.85 ± 0.69	<0.001^a^,^b^
**WMHV, ratio of ICV × 10^3^**	1.46 ± 1.55	3.05 ± 2.89	3.67 ± 3.64	<0.001^a^,^b^
**ICV, mL**	1364.96 ± 136.80	1348.92 ± 117.93	1277.96 ± 124.96	<0.001^b^,^c^
**^18^F-FBB PET scan**				
**Frontal SUVRs**	0.98 ± 0.10	1.46 ± 0.19	1.52 ± 0.20	<0.001^a^,^b^,^c^
**Parietal SUVRs**	1.02 ± 0.11	1.47 ± 0.18	1.53 ± 0.19	<0.001^a^,^b^,^c^
**Lateral temporal SUVRs**	0.96 ± 0.09	1.37 ± 0.17	1.43 ± 0.19	<0.001^a^,^b^,^c^
**Anterior/posterior cingulate SUVRs**	1.03 ± 0.11	1.55 ± 0.20	1.61 ± 0.20	<0.001^a^,^b^
**Global SUVRs**	0.99 ± 0.10	1.45 ± 0.18	1.51 ± 0.18	<0.001^a^,^b^,^c^

Values are expressed as mean ± standard deviation or number (percentage). *P* values are the results of analyses of variance, chi-square tests or Fisher’s Exact tests, as appropriate.

a Significantly different in comparison between normal control and AD non-dementia group.

b Significantly different in comparison between normal control and AD dementia group.

c Significantly different in comparison between AD non-dementia and dementia groups AD = Alzheimer’s disease; CDR-SOB = Clinical Dementia Rating Scale-Sum of boxes; CPV = choroid plexus volume; ICV = intracranial volume; LVV = lateral ventricle volume; MMSE = mini-mental status examination; MRI = magnetic resonance imaging; SUVR = standardized uptake value ratio; WMHV = white matter hyperintensity volume.

## RESULTS

### Demographic characteristics of the study participants

The mean age of patients with AD continuum at the time of MRI (n = 203) was 75.46 ± 7.06 years. Among them, 126 (62.1%) were women and 106 (52.2%) were APOE ε4 carriers (Supplementary Table 1). The demographic characteristics of the control (n = 82), AD non-dementia (n = 126), and AD dementia (n = 77) groups are summarized in Table 1. The mean age at the time of MRI was higher in the AD group than in the control group, but the proportion of male/female patients was comparable between the groups. Among the vascular risk factors, hypertension and diabetes mellitus were more prevalent in the AD group than those in the control group.

### Neuropsychological and imaging characteristics of the participants

**Figure 2.**
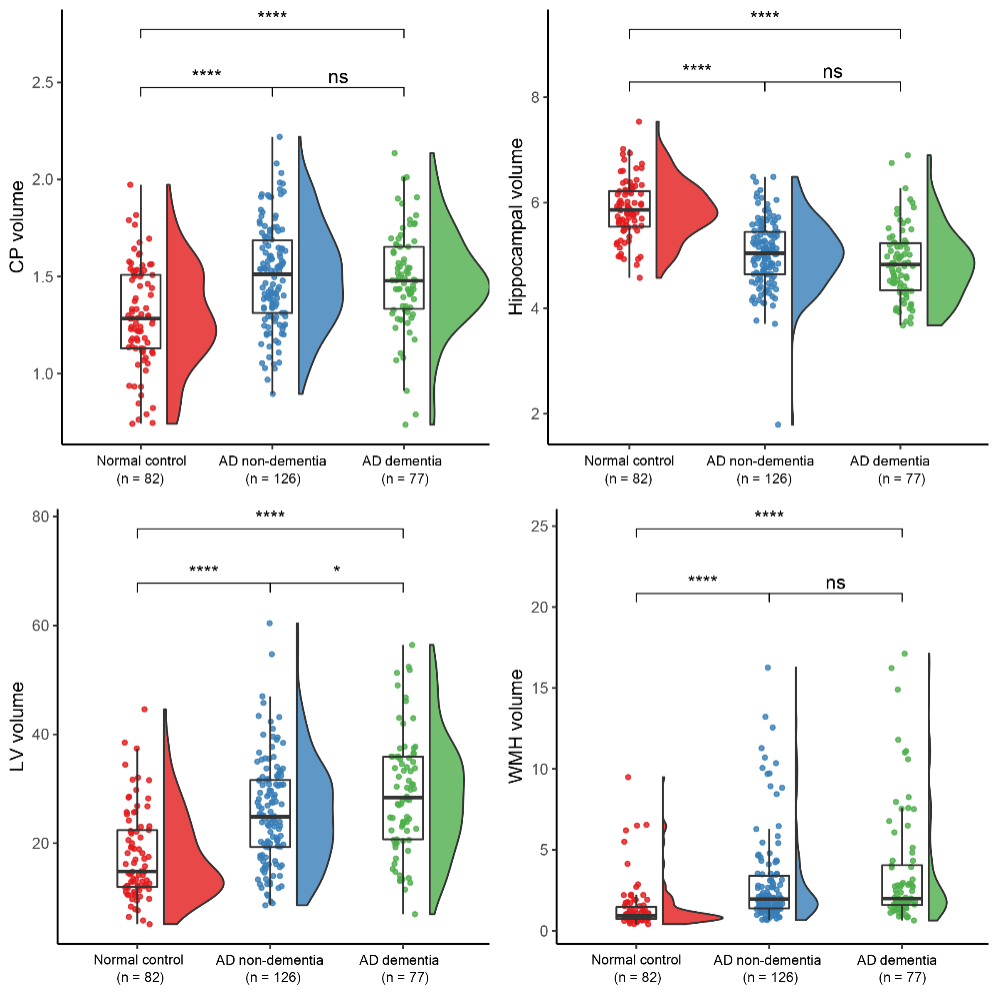
**Rain-cloud plots of MR volumetric imaging markers in the normal control, non-dementia AD, and dementia AD groups**. MR = magnetic resonance; AD = Alzheimer's disease; CP = choroid plexus; LV = lateral ventricle; WMH = white matter hyperintensity. *Uncorrected *P* < 0.05; ** uncorrected *P* < 0.01; *** uncorrected *P* < 0.001; **** uncorrected *P* < 0.0001.

The neuropsychological and imaging characteristics of the three groups are summarized in Table 1. As expected, the MMSE, CDR-SOB, and composite scores of each cognitive function domain were the worst in the AD dementia group. In terms of Aβ burden, all the global and regional SUVRs, except for the anterior/posterior cingulate SUVRs, were greater in the AD dementia group than those in the AD non-dementia group. The mean Dice score for the automatically segmented CP was 0.94 (standard deviation=0.04, range 0.83-0.99), indicating excellent accuracy compared to that of manual segmentation (the reference standard). The ICV was smaller in the AD dementia group than those in the AD non-dementia and control groups. CPV, LVV, and WMHV were all greater, while the hippocampal volume was smaller in the AD group than those in the control group. Within the AD group, the CPV, hippocampal volume, and WMHV were comparable between the two groups, whereas the LVV was greater in the AD dementia group than in the AD non-dementia group (Fig. 2). Correlation analyses revealed that the CPV was significantly associated with LVV (*r* = 0.44, *P* < 0.001), hippocampal volume (*r* = -0.30, *P* < 0.001), and WMHV (*r* = 0.22, *P* < 0.001).

### Relationship between CPV and Aβ burden

**Figure 3.**
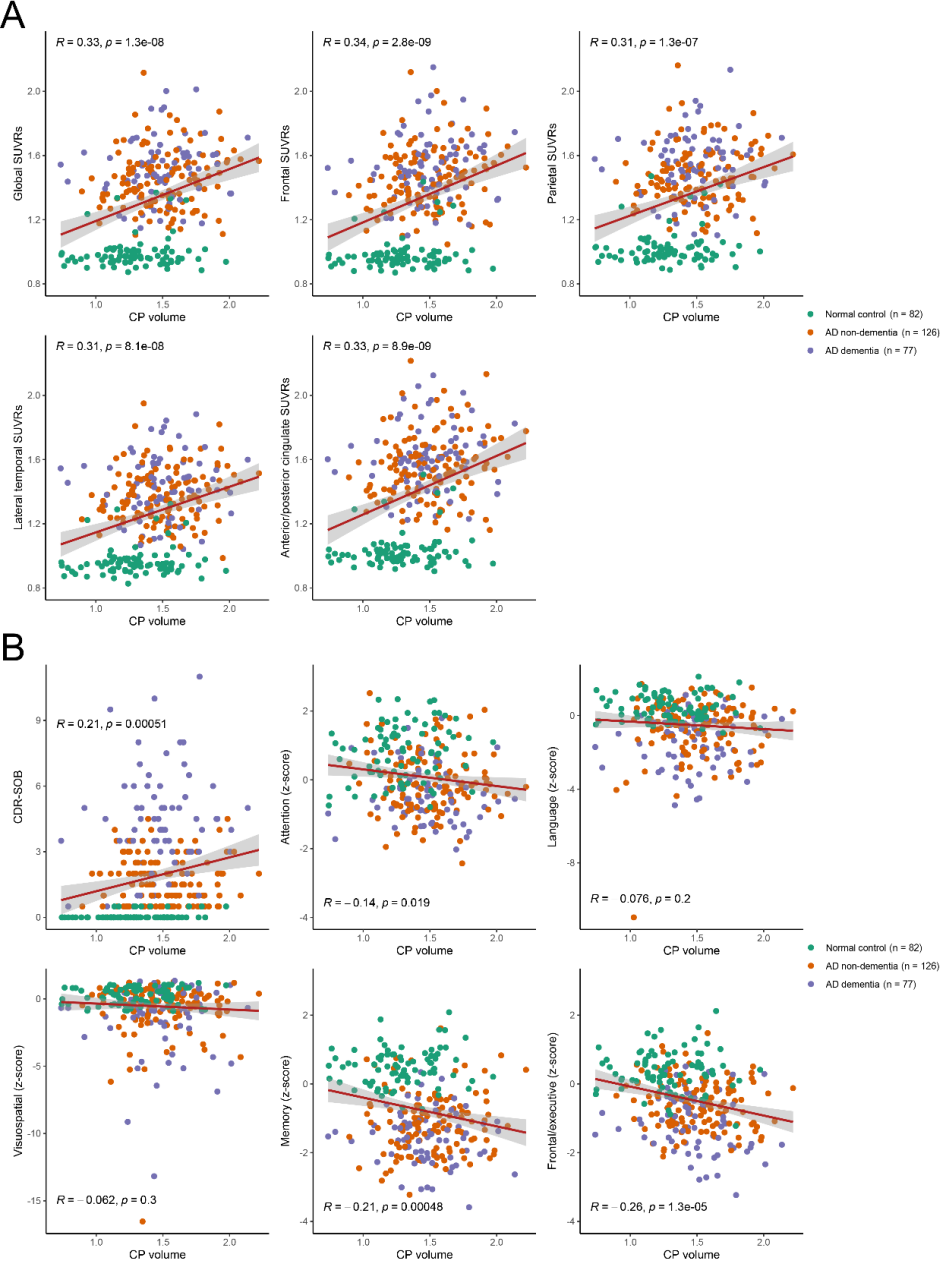
**Scatter plot of CP volume and amyloid burden (**A**) or cognitive function (**B**) in all participants (normal control, non-dementia AD, and dementia AD groups)**. CP = choroid plexus

Correlation analyses revealed that the CPV was significantly associated with the global (*r* = 0.33, *P* < 0.001) and regional (frontal [*r* = 0.34, *P* < 0.001], parietal [*r* = 0.31, *P* < 0.001], lateral temporal [*r* = 0.31, *P* < 0.001], and anterior/posterior cingulate [*r* = 0.33, *P* < 0.001]) SUVRs, as observed on ^18^F-FBB PET scans (Figure 3A). Multivariate linear regression analysis demonstrated that CPV was significantly associated with the global (*β* = 0.20, standard error [SE] = 0.07, *P* < 0.001) and regional (frontal [*β* = 0.22, SE = 0.07, *P* = 0.001, FDR-corrected *P =* 0.003], parietal [*β* = 0.19, SE = 0.07, *P* = 0.004, FDR-corrected *P =* 0.005], lateral temporal [*β* = 0.16, SE = 0.06, *P* = 0.008, FDR-corrected *P =* 0.008], and anterior/posterior cingulate [*β* = 0.24, SE = 0.07, *P* = 0.001, FDR-corrected *P =* 0.003]) SUVRs after adjusting for age and sex (Table 2). In the subgroup analysis, CPV was associated with global (*β* = 0.12, SE = 0.06, *P* = 0.027) and regional (frontal [*β* = 0.14, SE = 0.06, *P* = 0.016, FDR-corrected *P =* 0.048] and anterior/posterior cingulate [*β* = 0.14, SE = 0.06, *P* = 0.024, FDR-corrected *P =* 0.048]) SUVRs in the AD continuum group. The global SUVRs were associated with CPV in the AD non-dementia group (*β* = 0.16, SE = 0.07, *P* = 0.029), while no significant association was observed between CPV and global Aβ burden in the AD dementia group (*β* = 0.09, SE = 0.09, *P* = 0.331; Supplementary Table 2).

**Table 2:** Multivariate linear regression analyses for the effects of CPV on Aβ burden in all participants (normal control, non-dementia AD, and dementia AD groups).

	Frontal SUVRs*		Lateral Parietal SUVRs*		Lateral temporal SUVRs*		Anterior/posterior cingulate SUVRs*		Global SUVRs	
**Variables**	*β (SE)*	*P*	*β (SE)*	*P*	*β (SE)*	*P*	*β (SE)*	*P*	*β (SE)*	*P*
**Intercept**	0.23 (0.14)	0.101	0.30 (0.13)	0.026	0.27 (0.12)	0.028	0.28 (0.15)	0.059	0.26 (0.13)	0.047
**Age at MRI scan**	0.01 (0.00)	<0.001	0.01 (0.00)	<0.001	0.01 (0.00)	<0.001	0.01 (0.00)	<0.001	0.01 (0.00)	<0.001
**Female**	0.08 (0.03)	0.016	0.10 (0.03)	0.001	0.08 (0.03)	0.004	0.08 (0.03)	0.019	0.09 (0.03)	0.004
**CPV (ratio of ICV)**	0.22 (0.07)	0.001	0.19 (0.07)	0.004	0.16 (0.06)	0.008	0.24 (0.07)	0.001	0.20 (0.06)	0.002

Multivariate linear regression models were used to investigate the association between CPV and Aβ burden after adjusting for age at MRI scan and sex.

* FDR correction was applied for multiple comparison of four regional SUVRs (frontal, lateral parietal, lateral temporal, anterior/posterior cingulate) Bold indicates *P* or FDR-corrected *P* < 0.05. CPV = choroid plexus volume; ICV = intracranial volume; SE = standard error; SUVR = standardized uptake value ratio

### Relationship between CPV and cognitive function

In simple correlation analyses, CPV was significantly associated with the composite scores of the attention/working memory (*r* = -0.14, *P* = 0.019), memory (*r* = -0.21, *P* < 0.001), and frontal/executive function (*r* = -0.26, *P* < 0.001) domains, as well as CDR-SOB (*r* = 0.21, *P* < 0.001; Figure 3B). After adjusting for covariates, the multivariate linear regression model revealed a significant association between CPV and CDR-SOB (*β* = 0.85, SE = 0.40, *P* = 0.034), composite scores of memory (*β* = -0.65, SE = 0.23, *P* = 0.005, FDR-corrected *P =* 0.009), and frontal/executive function (*β* = -0.68, SE = 0.18, *P* < 0.001, FDR-corrected *P <* 0.001) domains (Table 3). In the subgroup analysis, although a trend was observed towards an association between CPV and CDR-SOB (*β* = 0.70, SE = 0.42, *P* = 0.096) in the AD non-dementia group, the other models demonstrated no association between CPV and the composite scores of cognitive function domains (Supplementary Table 3).

**Table 3:** Multivariate linear regression analyses for the effects of CPV on cognitive function in all participants (normal control, non-dementia AD, and dementia AD groups).

	Attention*		Language*		Visuospatial*		Memory*		Frontal/executive*		CDR-SOB	
**Variables**	*β (SE)*	*P*	*β (SE)*	*P*	*β (SE)*	*P*	*β (SE)*	*P*	*β (SE)*	*P*	*β (SE)*	*P*
**Intercept**	1.88 (0.39)	<0.001	2.06 (0.60)	0.001	1.28 (0.83)	0.125	1.44 (0.43)	0.001	1.33 (0.34)	<0.001	-4.51 (1.00)	<0.001
**Age at MRI scan**	-	-	-	-	-	-	-	-	-	-	0.02 (0.01)	0.149
**Female**	-0.20 (0.11)	0.085	-0.44 (0.18)	0.013	-0.08 (0.24)	0.730	0.04 (0.13)	0.779	0.18 (0.10)	0.067	0.27 (0.20)	0.173
**Education**	-	-	-	-	-	-	-	-	-	-	-0.03 (0.02)	0.155
**Cognitive status**												
**Non-dementia**	Ref.	-	Ref.	-	Ref.	-	Ref.	-	Ref.	-	Ref.	-
**Dementia**	-0.63 (0.13)	<0.001	-0.98 (0.19)	<0.001	-0.95 (0.27)	0.001	-1.02 (0.14)	<0.001	-0.9 (0.11)	<0.001	2.94 (0.21)	0.000
**CPV (ratio of ICV)**	-0.47 (0.20)	0.020	-0.44 (0.31)	0.159	-0.34 (0.43)	0.428	-0.68 (0.22)	0.002	-0.65 (0.18)	<0.001	0.85 (0.40)	0.034

Multivariate linear regression models were used to investigate the association between CPV and five cognitive domains after adjusting for sex and cognitive status. In terms of the model for CDR-SOB, age at MRI scan and years of education were additionally adjusted.

* FDR correction was applied for multiple comparison of five cognitive domains (attention, language, visuospatial, memory, and frontal/executive domains) Bold indicates *P* or FDR-corrected *P* < 0.05. CDR-SOB = Clinical Dementia Rating Scale-Sum of boxes; CPV = choroid plexus volume; MRI = magnetic resonance imaging; SE = standard error.

### Longitudinal assessment of the changes in MMSE scores according to CPV

A subsample of 147 patients on the AD continuum who underwent serial MMSE at least twice with one year interval exhibited demographic and clinical characteristics similar to those of all participants in the present study (Supplementary Table 4). The demographic characteristics of the AD non-dementia group (n = 94) and the AD dementia group (n = 53) are provided in Supplementary Table 5. In the 147 patients on the AD continuum, the CPV × time interaction term was not significant in the linear mixed model, indicating that longitudinal decreases in MMSE scores were not affected by CPV (*β* = 0.00, SE = 0.10, *P* = 0.983). In subgroup analyses based on cognitive status, the CPV × time interaction term was significant only in the AD dementia subgroup (*β* = -0.58, SE = 0.20, *P* = 0.004) but not in the AD non-dementia subgroup (*β* = 0.18, SE = 0.12, *P* = 0.137; Table 4), indicating that a larger CPV was associated with a more rapid decline in MMSE scores in the AD dementia subgroup.

**Table 4:** Longitudinal models for the effect of CPV on MMSE score change over time in the AD group.

	*All patients*		*AD non-dementia subgroup*		*AD Dementia subgroup*	
	*β* (SE)	*p*	*β* (SE)	*p*	*β* (SE)	*p*
**Intercept**	26.47 (3.77)	<0.001	24.14 (4.40)	<0.001	20.52 (6.69)	0.004
**Age at MRI scan**	0.03 (0.04)	0.381	0.01 (0.04)	0.850	0.05 (0.07)	0.439
**Female**	-0.62 (0.53)	0.245	-0.04 (0.60)	0.941	-1.21 (1.01)	0.238
**Education**	0.33 (0.05)	<0.001	0.26 (0.06)	0.000	0.35 (0.09)	0.001
***APOE* ε4 carrier**	-0.63 (0.47)	0.182	0.01 (0.53)	0.992	-1.67 (0.93)	0.080
**Cognitive status**						
**Non-dementia**	Ref.	-	-	-	-	-
**Dementia**	-3.17 (0.51)	<0.001	-	-	-	-
**Global Aβ SUVRs**	-2.13 (1.45)	0.144	-1.88 (1.65)	0.256	-2.31 (2.67)	0.390
**CPV (ratio of ICV)**	-0.47 (0.28)	0.096	-0.18 (0.33)	0.592	-0.66 (0.50)	0.194
**Time**	-1.40 (0.11)	<0.001	-1.24 (0.14)	<0.001	-1.87 (0.19)	<0.001
**CPV × time**	0.00 (0.10)	0.983	0.18 (0.12)	0.137	-0.58 (0.20)	0.004

Results of linear mixed model for MMSE score change over time after adjusting for age at MRI scan, sex, years of education, *APOE* ε4 carrier status, cognitive status, global Aβ SUVRs, CPV, time, and CPV × time. Aβ, global standardized uptake value ratios; *β*, regression coefficient; CPV, choroid plexus volume; ICV, intracranial volume; MMSE, Mini-Mental Status Examination; SE, standard error; SUVRs, standardized uptake value ratios.

## DISCUSSION

In the present study, we investigated the relationship between CPV, Aβ deposition, and cognitive function in patients on the AD continuum. The major findings were as follows: (1) CPV was larger in patients on the AD continuum than in healthy controls, whereas no difference was observed in CPV between the AD non-dementia and AD dementia groups; (2) a larger CPV was associated with higher baseline global and regional Aβ deposition; (3) a larger CPV was associated with worse general cognition and poorer performance on memory and frontal/executive function domains; and (4) a larger CPV was associated with a more rapid decline in MMSE scores in the AD dementia group, but not in the AD non-dementia group. These findings suggest that CP enlargement is observed in patients on the AD continuum and is associated with increased Aβ deposition, which is regarded as a crucial factor in the pathogenesis of AD.

Previous research has suggested that the CP can be a promising therapeutic target for neurodegenerative disorders (i.e., protein misfolding diseases, such as AD and Parkinson’s disease) owing to its primary role in controlling the production and composition of CSF and the fact that harmful metabolites in the brain are eliminated through the CSF [32]. Although the specific relationship between changes in the morphology or volume in the CP and neurological disorders remains unclear, recent studies have shown that an increase in CPV is linked to poor cognitive performance in patients on the AD continuum [12, 13]. Additionally, various preclinical studies have demonstrated that CP function influences the clearance of Aβ from the brain in an AD mouse model [8, 9]. The differences in CPV between the AD and control groups in the present study are in line with the findings reported in previous studies [12-14]; however, the lack of any difference in CPV between the AD non-dementia and AD dementia groups contradicts the results of previous studies [13]. Although a previous study enrolled patients with AD based on clinical diagnosis, the rate of Aβ positivity was less than 70% in study participants. In contrast, the present study enrolled patients on the AD continuum who exhibited Aβ positivity on ^18^F-FBB PET scans. Considering that Aβ positivity is major determinant for the diagnosis of AD continuum [33], the enrollment of patients with non-AD pathology in the previous study [13] may be attributed to contrasting results. Another possible explanation for our findings (i.e., a significant difference in CPV between patients on the AD continuum and healthy controls, but no difference between the AD subgroups) is that Aβ deposition primarily occurs in the early stages of AD and CP enlargement may primarily affect Aβ deposition during the transition from a cognitively normal status to prodromal AD.

In the present study, multivariate linear regression analyses demonstrated that the global and regional Aβ burden were positively associated with CPV. These findings were not in accordance with those of previous studies, which failed to demonstrate the relationship between CPV and Aβ deposition [13, 14]. The discrepancy between these findings may be due to the differences in the study design and availability of Aβ PET scans or quantitative analyses. Choi *et al.* investigated only the binary results from Aβ PET (either Aβ positivity or negativity), while we calculated the detailed SUVRs from each subregion of the brain and analyzed the relationship between the CPV and Aβ deposition. As a result, Choi *et al.* reported that there was no significant correlation between CPV and Aβ positivity, whereas we found that the global and regional Aβ burden were positively associated with CPV [13]. Considering that CP is closely related to glymphatic function [34], the results of previous preclinical and clinical studies exploring the role of glymphatic dysfunction in the pathogenesis of AD may provide possible explanations for our findings [35, 36]. The deletion of aquaporin-4 water channels significantly enhances the accumulation of Aß plaques and cognitive impairments by inhibiting the glymphatic flow, as observed in a mouse model of AD [37]. Similarly, in humans, individuals with AD exhibited reduced drainage of CSF containing Aß and phosphorylated tau compared to that of age-matched healthy controls [38]. Conversely, a previous study demonstrated the suppressive effect of Aß overexpression on glymphatic activity [39]. In subgroup analysis, the global SUVRs were associated with CPV only in the AD non-dementia group, and not in the AD dementia group. Although Aβ aggregates along with cognitive decline in AD, Aβ burden is known to be saturated in dementia state [40]. The lack of association between Aβ burden and CPV in the AD dementia group may be attributed to the ceiling effect. This is the first study to demonstrate the relationship between CPV and Aß deposition. Considering that the initial event which triggers neuronal degradation in Alzheimer’s disease is enhanced Aß generation and aggregation [41], CP function would be an important therapeutic target for the prevention of AD development.

CP enlargement is believed to be a reflection of CP dysfunction, which could interfere with the effective removal of waste products from the brain via the glymphatic system. Therefore, an increase in CPV could represent a condition in which neurodegenerative processes are facilitated in patients on the AD continuum. However, our previous study did not find an association between the number of enlarged perivascular spaces (EPVS) in the basal ganglia, centrum semiovale, or hippocampus, which is regarded as an imaging marker of glymphatic dysfunction, and Aβ burden in patients on the AD continuum [42]. Nevertheless, the accuracy of visual rating of EPVS is limited. Further studies using the volumetry of EPVS derived from 3D high-resolution T2 images are required that may yield different results.

In the present study, we investigated the association between CPV and general cognition and performance in each cognitive function domain. Multivariate linear regression analyses demonstrated that a larger CPV was closely related to poorer general cognition, as assessed by CDR-SOB, and more severely impaired memory and frontal/executive functions. Memory function is the earliest and most frequently affected neurocognitive domain in patients with AD, and an association between enlarged CP and poor memory function is plausible. Regarding frontal/executive function, a previous study demonstrated that neocortical tau deposition was associated with an accelerated decline in executive function over time in patients with AD [43]. The association between large CPV and poor frontal/executive function may be attributed to the fact that CP enlargement could affect widespread tau deposition. However, we were unable to investigate this relationship in the present study due to a lack of data on tau biomarkers. Therefore, further studies are warranted to demonstrate the relationships among CP dysfunction, tau deposition, and cognitive function.

A linear mixed model revealed that a larger CPV was associated with a more rapid decline in MMSE scores in the AD dementia subgroup than that of the AD non-dementia subgroup, suggesting a possible effect of CPV on the longitudinal cognitive prognosis in patients on the AD continuum. However, we did not identify any association between CPV and the rate of longitudinal decline in MMSE scores over time in the non-dementia subgroup. When considering a possible explanation for the cognitive status-dependent effect of CPV on cognitive decline, multiple factors associated with cognitive reserve may work during the AD non-dementia stage; however, cognitive decline in the advanced AD stage is much faster once the critical threshold is reached. In addition, because the MMSE has limitations in measuring subtle cognitive decline in early AD owing to a large measurement error and substantial variation in the annual score [44], further studies using serial neuropsychological tests are required to demonstrate the exact relationship between CPV and cognitive outcomes in AD.

Our study findings support the close relationship between the dysfunction of glymphatic system indicated by enlarged CP and Aβ, which broadens our understanding of AD pathogenesis in the context of glymphatic system. We also observed that as CPV increased, the patients on AD continuum are more likely to have worse cognitive function and show a more rapid decrease in MMSE scores during follow-up. Therefore, enlarged CP may allow for risk stratification for future cognitive decline, which can guide individualized follow-up strategies. For instance, patients with a large baseline CPV might undergo more intensive follow-up to assess the likelihood of early conversion to dementia or can be candidates for active treatment. As the CPV can be relatively easily and automatically obtained from the baseline MRI, it can be a feasible prognostic imaging biomarker which can be integrated in clinical practice for AD patients. Future studies including large amount of normative data and serial neuropsychological tests for longitudinal analysis are warranted to validate the prognostic role of CPV.

Accurate segmentation of the CP from high-resolution structural MRI is a prerequisite for investigating the clinical relevance of CP. Manual segmentation has been the gold standard for CP segmentation, and using contrast-enhanced images enables a better visualization of CP than using non-contrast images, as the fenestrated endothelium in CP allows contrast to accumulate in the interstitium [45]. However, contrast agents is not routinely used in both clinical and research settings, instead non-contrast high-resolution 3D T1-weighted images are usually included in baseline MRI scans. Therefore, an accurate segmentation of the CP using non-contrast images is highly desirable. Furthermore, because manual segmentation is a time-consuming and labor-intensive procedure, it is not practical for investigating CP in a large number of patients. Several previous studies have performed automatic CP segmentation using non-contrast 3D T1-weighted with FreeSurfer software [46-48] and have reported an acceptable accuracy of automatic CP segmentation. In the present study, we utilized FastSurfer, a fast pipeline for the neuroanatomical surfaces, which has been reported to outperform FreeSurfer with respect to runtime, reliability, and sensitivity [22]. This approach provides a full FreeSurfer alternative for volumetric analysis in markedly reduced time, while maintaining segmentation accuracy and test-retest reliability, making it more feasible than FreeSurfer [22, 49]. In our study, we observed the excellent accuracy of automatic CP segmentation results from FastSurfer as compared to those of manual segmentation. Therefore, we believe that automatic segmentation of the CP using non-contrast T1-weighted images with FastSurfer is a reliable and accurate method for CP segmentation.

Our study had some limitations. First, this was a retrospective cross-sectional study; as such, causal relationships could not be demonstrated. Also, the relatively small sample size, particularly in the AD dementia group, may have affected the statistical power and generalizability of the findings. Further longitudinal comparative studies using sequential neuropsychological test, brain MRI, and Aβ PET imaging will be required to elucidate these relationships. Second, the relatively small sample size of the AD subgroups may have led to statistically insignificant results in the sensitivity analyses performed separately in the AD non-dementia and AD dementia groups, although the floor or ceiling effect in the AD group may have interfered with the correlation of clinical and imaging measures. Third, tau protein, which is another misfolded protein involved in AD pathogenesis, was not considered in the present study. We hope that future studies will demonstrate the relationship between CP function and tau protein levels in patients on the AD continuum.

In conclusion, our study demonstrated that CP enlargement was observed in patients on the AD continuum, and that it was associated with increased Aβ deposition and impaired memory and frontal/executive function in these patients. Furthermore, a larger CPV was associated with a more rapid decline in MMSE scores in the AD dementia group. Overall, these findings suggest that CP is closely related to AD pathogenesis and could be considered as a therapeutic target in AD.

## Supplementary Materials

The Supplementary data can be found online at: www.aginganddisease.org/EN/10.14336/AD.2024.0118.

Data generated or analyzed during the study are available from the corresponding author by request.



## References

[1] DuyckaertsC, DelatourB, PotierM-C (2009). Classification and basic pathology of Alzheimer disease. Acta Neuropathologica, 118:5-36.19381658 10.1007/s00401-009-0532-1

[2] MawuenyegaKG, SigurdsonW, OvodV, MunsellL, KastenT, MorrisJC, et al. (2010). Decreased clearance of CNS beta-amyloid in Alzheimer's disease. Science, 330:1774.21148344 10.1126/science.1197623PMC3073454

[3] Tarasoff-ConwayJM, CarareRO, OsorioRS, GlodzikL, ButlerT, FieremansE, et al. (2015). Clearance systems in the brain-implications for Alzheimer disease. Nat Rev Neurol, 11:457-470.26195256 10.1038/nrneurol.2015.119PMC4694579

[4] HonigLS, VellasB, WoodwardM, BoadaM, BullockR, BorrieM, et al. (2018). Trial of Solanezumab for Mild Dementia Due to Alzheimer’s Disease. New England Journal of Medicine, 378:321-330.29365294 10.1056/NEJMoa1705971

[5] SallowayS, SperlingR, FoxNC, BlennowK, KlunkW, RaskindM, et al. (2014). Two Phase 3 Trials of Bapineuzumab in Mild-to-Moderate Alzheimer's Disease. New England Journal of Medicine, 370:322-333.24450891 10.1056/NEJMoa1304839PMC4159618

[6] MintunMA, LoAC, Duggan EvansC, WesselsAM, ArdayfioPA, AndersenSW, et al. (2021). Donanemab in Early Alzheimer’s Disease. New England Journal of Medicine, 384:1691-1704.33720637 10.1056/NEJMoa2100708

[7] van DyckCH, SwansonCJ, AisenP, BatemanRJ, ChenC, GeeM, et al. (2022). Lecanemab in Early Alzheimer’s Disease. New England Journal of Medicine, 388:9-21.36449413 10.1056/NEJMoa2212948

[8] CrossgroveJS, LiGJ, ZhengW (2005). The choroid plexus removes beta-amyloid from brain cerebrospinal fluid. Exp Biol Med (Maywood), 230:771-776.16246905 10.1177/153537020523001011PMC3982214

[9] González-MarreroI, Giménez-LlortL, JohansonCE, Carmona-CaleroEM, Castañeyra-RuizL, Brito-ArmasJM, et al. (2015). Choroid plexus dysfunction impairs beta-amyloid clearance in a triple transgenic mouse model of Alzheimer's disease. Front Cell Neurosci, 9:17.25705176 10.3389/fncel.2015.00017PMC4319477

[10] SerotJM, BénéMC, FaureGC (2003). Choroid plexus, aging of the brain, and Alzheimer's disease. Front Biosci, 8:s515-521.12700093 10.2741/1085

[11] SerotJ-M, BénéM-C, FoliguetB, FaureGC (2000). Morphological alterations of the choroid plexus in late-onset Alzheimer’s disease. Acta Neuropathologica, 99:105-108.10672315 10.1007/pl00007412

[12] Čarna M, Onyango IG, Katina S, Holub D, Novotny JS, Nezvedova M, et al. (2023). Pathogenesis of Alzheimer's disease: Involvement of the choroid plexus. Alzheimers Dement. 10.1002/alz.12970 PMC10634590 36825691

[13] ChoiJD, MoonY, KimHJ, YimY, LeeS, MoonWJ (2022). Choroid Plexus Volume and Permeability at Brain MRI within the Alzheimer Disease Clinical Spectrum. Radiology, 304:635-645.35579521 10.1148/radiol.212400

[14] TadayonE, Pascual-LeoneA, PressD, SantarnecchiE (2020). Choroid plexus volume is associated with levels of CSF proteins: relevance for Alzheimer's and Parkinson's disease. Neurobiol Aging, 89:108-117.32107064 10.1016/j.neurobiolaging.2020.01.005PMC9094632

[15] Jeong SH, Cha J, Park M, Jung JH, Ye BS, Sohn YH, et al. (2022). Association of Enlarged Perivascular Spaces With Amyloid Burden and Cognitive Decline in Alzheimer Disease Continuum. Neurology, 10.1212/WNL.0000000000200989. 35985826

[16] DuboisB, HampelH, FeldmanHH, ScheltensP, AisenP, AndrieuS, et al. (2016). Preclinical Alzheimer's disease: Definition, natural history, and diagnostic criteria. Alzheimers Dement, 12:292-323.27012484 10.1016/j.jalz.2016.02.002PMC6417794

[17] AlbertMS, DeKoskyST, DicksonD, DuboisB, FeldmanHH, FoxNC, et al. (2011). The diagnosis of mild cognitive impairment due to Alzheimer's disease: recommendations from the National Institute on Aging-Alzheimer's Association workgroups on diagnostic guidelines for Alzheimer's disease. Alzheimers Dement, 7:270-279.21514249 10.1016/j.jalz.2011.03.008PMC3312027

[18] McKhannGM, KnopmanDS, ChertkowH, HymanBT, JackCRJr., KawasCH, et al. (2011). The diagnosis of dementia due to Alzheimer's disease: recommendations from the National Institute on Aging-Alzheimer's Association workgroups on diagnostic guidelines for Alzheimer's disease. Alzheimers Dement, 7:263-269.21514250 10.1016/j.jalz.2011.03.005PMC3312024

[19] BarthelH, GertzHJ, DreselS, PetersO, BartensteinP, BuergerK, et al. (2011). Cerebral amyloid-β PET with florbetaben (18F) in patients with Alzheimer's disease and healthy controls: a multicentre phase 2 diagnostic study. Lancet Neurol, 10:424-435.21481640 10.1016/S1474-4422(11)70077-1

[20] JagustWJ, LandauSM, KoeppeRA, ReimanEM, ChenK, MathisCA, et al. (2015). The Alzheimer's Disease Neuroimaging Initiative 2 PET Core: 2015. Alzheimers Dement, 11:757-771.26194311 10.1016/j.jalz.2015.05.001PMC4510459

[21] MorminoEC, KluthJT, MadisonCM, RabinoviciGD, BakerSL, MillerBL, et al. (2009). Episodic memory loss is related to hippocampal-mediated beta-amyloid deposition in elderly subjects. Brain, 132:1310-1323.19042931 10.1093/brain/awn320PMC2677792

[22] HenschelL, ConjetiS, EstradaS, DiersK, FischlB, ReuterM (2020). FastSurfer - A fast and accurate deep learning based neuroimaging pipeline. Neuroimage, 219:117012.32526386 10.1016/j.neuroimage.2020.117012PMC7898243

[23] ZouKH, WarfieldSK, BharathaA, TempanyCM, KausMR, HakerSJ, et al. (2004). Statistical validation of image segmentation quality based on a spatial overlap index1: scientific reports. Academic radiology, 11:178-189.14974593 10.1016/S1076-6332(03)00671-8PMC1415224

[24] Kang Y, Jahng S, Na D (2012). Seoul Neuropsychological Screening Battery. (SNSB-II): Professional Manual. Incheon: Human Brain Research and Consulting.

[25] RyuHJ, YangDW (2023). The Seoul Neuropsychological Screening Battery (SNSB) for Comprehensive Neuropsychological Assessment. Dement Neurocogn Disord, 22:1-15.36814700 10.12779/dnd.2023.22.1.1PMC9939572

[26] JangH, YeBS, WooS, KimSW, ChinJ, ChoiSH, et al. (2017). Prediction Model of Conversion to Dementia Risk in Subjects with Amnestic Mild Cognitive Impairment: A Longitudinal, Multi-Center Clinic-Based Study. J Alzheimers Dis, 60:1579-1587.28968237 10.3233/JAD-170507

[27] (1997). A validity study on the korean mini-mental state examination (K-MMSE) in dementia patients. J Korean Neurol Assoc, 15:300-308.

[28] Ku HM, Kim JH, Lee HS, Ko HJ, Kwon EJ, Jo S, et al. (2004). A Study on the Reliability and Validity of Seoul-Activities of Daily Living(S-ADL). Ann Geriatr Med Res, 8:206-214.

[29] (2002). "The Reliability and Validity of the Korean Instrumental Activities of Daily Living (K-IADL)". J Korean Neurol Assoc, 20:8-14.

[30] LaoPJ, BetthauserTJ, HillmerAT, PriceJC, KlunkWE, MihailaI, et al. (2016). The effects of normal aging on amyloid-β deposition in nondemented adults with Down syndrome as imaged by carbon 11-labeled Pittsburgh compound B. Alzheimers Dement, 12:380-390.26079411 10.1016/j.jalz.2015.05.013PMC4677061

[31] Saunders TS, Jenkins N, Blennow K, Ritchie C, Muniz-Terrera G (2022). Interactions between apolipoprotein E, sex, and amyloid-beta on cerebrospinal fluid p-tau levels in the European prevention of Alzheimer's dementia longitudinal cohort study (EPAD LCS). eBioMedicine, 83. 10.1016/j.ebiom.2022.104241 PMC9440380 36041266

[32] MunicioC, CarreroL, AntequeraD, CarroE (2023). Choroid Plexus Aquaporins in CSF Homeostasis and the Glymphatic System: Their Relevance for Alzheimer's Disease. Int J Mol Sci, 24.36614315 10.3390/ijms24010878PMC9821203

[33] JackCRJr, BennettDA, BlennowK, CarrilloMC, DunnB, HaeberleinSB, et al. (2018). NIA-AA Research Framework: Toward a biological definition of Alzheimer's disease. Alzheimers Dement, 14:535-562.29653606 10.1016/j.jalz.2018.02.018PMC5958625

[34] ChristensenJ, LiC, MychasiukR (2022). Choroid plexus function in neurological homeostasis and disorders: The awakening of the circadian clocks and orexins. J Cereb Blood Flow Metab, 42:1163-1175.35296175 10.1177/0271678X221082786PMC9207490

[35] ReevesBC, KarimyJK, KundishoraAJ, MestreH, CerciHM, MatoukC, et al. (2020). Glymphatic System Impairment in Alzheimer's Disease and Idiopathic Normal Pressure Hydrocephalus. Trends Mol Med, 26:285-295.31959516 10.1016/j.molmed.2019.11.008PMC7489754

[36] NedergaardM, GoldmanSA (2020). Glymphatic failure as a final common pathway to dementia. Science, 370:50-56.33004510 10.1126/science.abb8739PMC8186542

[37] XuZ, XiaoN, ChenY, HuangH, MarshallC, GaoJ, et al. (2015). Deletion of aquaporin-4 in APP/PS1 mice exacerbates brain Aβ accumulation and memory deficits. Mol Neurodegener, 10:58.26526066 10.1186/s13024-015-0056-1PMC4631089

[38] de LeonMJ, LiY, OkamuraN, TsuiWH, Saint-LouisLA, GlodzikL, et al. (2017). Cerebrospinal Fluid Clearance in Alzheimer Disease Measured with Dynamic PET. J Nucl Med, 58:1471-1476.28302766 10.2967/jnumed.116.187211PMC5577629

[39] PengW, AchariyarTM, LiB, LiaoY, MestreH, HitomiE, et al. (2016). Suppression of glymphatic fluid transport in a mouse model of Alzheimer's disease. Neurobiol Dis, 93:215-225.27234656 10.1016/j.nbd.2016.05.015PMC4980916

[40] BjorkliC, SandvigA, SandvigI (2020). Bridging the Gap Between Fluid Biomarkers for Alzheimer’s Disease, Model Systems, and Patients. Frontiers in Aging Neuroscience, 12.32982716 10.3389/fnagi.2020.00272PMC7492751

[41] JackCRJr., KnopmanDS, JagustWJ, ShawLM, AisenPS, WeinerMW, et al. (2010). Hypothetical model of dynamic biomarkers of the Alzheimer's pathological cascade. Lancet Neurol, 9:119-128.20083042 10.1016/S1474-4422(09)70299-6PMC2819840

[42] Jeong SH, Cha J, Park M, Jung JH, Ye BS, Sohn YH, et al. (2022). Association of Enlarged Perivascular Spaces With Amyloid Burden and Cognitive Decline in Alzheimer Disease Continuum. Neurology. 10.1212/WNL.0000000000200989 35985826

[43] Ossenkoppele R, Pichet Binette A, Groot C, Smith R, Strandberg O, Palmqvist S, et al. (2022). Amyloid and tau PET-positive cognitively unimpaired individuals are at high risk for future cognitive decline. Nature Medicine, 28:2381-2387. 10.1038/s41591-022-02049-x PMC9671808 36357681

[44] ClarkCM, SheppardL, FillenbaumGG, GalaskoD, MorrisJC, KossE, et al. (1999). Variability in annual Mini-Mental State Examination score in patients with probable Alzheimer disease: a clinical perspective of data from the Consortium to Establish a Registry for Alzheimer's Disease. Arch Neurol, 56:857-862.10404988 10.1001/archneur.56.7.857

[45] ShiY, LiX, ChenX, XuY, BoG, ZhouH, et al. (2017). Imaging findings of extraventricular choroid plexus papillomas: A study of 10 cases. Oncol Lett, 13:1479-1485.28454280 10.3892/ol.2016.5552PMC5403489

[46] ChoiJD, MoonY, KimH-J, YimY, LeeS, MoonW-J (2022). Choroid Plexus Volume and Permeability at Brain MRI within the Alzheimer Disease Clinical Spectrum. Radiology, 304:635-645.35579521 10.1148/radiol.212400

[47] RiciglianoVAG, MorenaE, ColombiA, ToniettoM, HamzaouiM, PoirionE, et al. (2021). Choroid Plexus Enlargement in Inflammatory Multiple Sclerosis: 3.0-T MRI and Translocator Protein PET Evaluation. Radiology, 301:166-177.34254858 10.1148/radiol.2021204426

[48] TadayonE, Pascual-LeoneA, PressD, SantarnecchiE (2020). Choroid plexus volume is associated with levels of CSF proteins: relevance for Alzheimer's and Parkinson's disease. Neurobiol Aging, 89:108-117.32107064 10.1016/j.neurobiolaging.2020.01.005PMC9094632

[49] Bloch L, Friedrich CM.2021. Comparison of Automated Volume Extraction with FreeSurfer and FastSurfer for Early Alzheimer's Disease Detection with Machine Learning. In 2021 IEEE 34th International Symposium on Computer-Based Medical Systems (CBMS). 113-118.

