# Supplementary material for: Choroid Plexus Volume, Amyloid Burden, and Cognition in the Alzheimer’s Disease Continuum
Source: Aging Dis. 2024 Jan 18;16(1):552–64. doi: 10.14336/AD.2024.0118 (PMC11745423; doi:10.14336/AD.2024.0118)
Supplement: Supplementary file 1 [file AD-16-1-552-s.pdf]

Choroid Plexus Volume, Amyloid Burden, and Cognition in the Alzheimer's Disease Continuum

Seong Ho Jeong, Chae Jung Park, Jungho Cha, Sang-Young Kim, Seung-Koo Lee, Yun Joong Kim, Young H. Sohn, Seok Jong Chung, Phil Hyu Lee

Supplementary Table 1. Demographic characteristics in patients on the AD continuum.

	AD continuum (n = 203)
**Demographic characteristics**	
Age at MRI scan	75.46 ± 7.06
Female, n (%)	126 (62.1%)
Education, y	10.74 ± 5.23
MMSE	23.04 ± 3.91
APOE ε4 carrier	106 (52.2%)
Cognitive status	
Non-dementia	126 (62.1%)
Dementia	77 (37.9%)
Vascular risk factors	
Hypertension	107 (52.7%)
Diabetes mellitus	44 (21.7%)
Dyslipidemia	59 (29.1%)
**Neuropsychological tests**	
Attention/working memory	-0.20 ± 0.87
Language	-0.92 ± 1.54
Visuospatial	-0.87 ± 2.25
Memory	-1.28 ± 0.86
Frontal/executive	-0.78 ± 0.80
CDR-SOB	-0.20 ± 0.87
**Volumetric MRI measures**	
CPV, ratio of ICV × 10^3	1.53 ± 0.34
LVV, ratio of ICV × 10^3	26.59 ± 7.98
hippocampal volume, ratio of ICV × 10^3	5.15 ± 1.30
WMHV, ratio of ICV × 10^3	3.16 ± 2.81
ICV, mL	1322.01 ± 125.19
**^{18}F-FBB PET scan**	
Frontal SUVRs	1.48 ± 0.19
Parietal SUVRs	1.49 ± 0.19
Lateral temporal SUVRs	1.39 ± 0.18
Anterior/posterior cingulate SUVRs	1.57 ± 0.20
Global SUVRs	1.47 ± 0.18

Values are expressed as mean ± standard deviation or number (percentage).
AD = Alzheimer's disease; CDR-SOB = Clinical Dementia Rating Scale-Sum of boxes; CPV = choroid plexus volume; ICV = intracranial volume; LVV = lateral ventricle volume; MMSE = mini-mental status examination; MRI = magnetic resonance imaging; SUVR = standardized uptake value ratio; WMHV = white matter hyperintensity volume

Supplementary Table 2. Multivariate linear regression analyses for the effects of CPV on amyloid burden in subgroup.

		Frontal SUVRs*		Lateral Parietal SUVRs*		Lateral temporal SUVRs*		Anterior/posterior cingulate SUVRs*		Global SUVRs	
	Variables	*β (SE)*	*P*	*β (SE)*	*P*	*β (SE)*	*P*	*β (SE)*	*P*	*β (SE)*	*P*
AD group	Intercept	1.21 (0.17)	<0.001	1.28 (0.16)	<0.001	1.18 (0.16)	<0.001	1.39 (0.17)	<0.001	1.25 (0.16)	<0.001
	Age	0.00 (0.00)	0.879	0.00 (0.00)	0.663	0.00 (0.00)	0.980	0.00 (0.00)	0.495	0.00 (0.00)	0.742
	Female	0.05 (0.03)	0.122	0.07 (0.03)	0.020	0.05 (0.03)	0.050	0.04 (0.03)	0.144	0.05 (0.03)	0.047
	APOE ε4	0.01 (0.03)	0.749	0.01 (0.03)	0.821	-0.03 (0.03)	0.308	0.02 (0.03)	0.447	0.00 (0.03)	0.957
	CPV	**0.14 (0.06)**	**0.016**	0.11 (0.06)	0.053	0.09 (0.05)	0.098	**0.14 (0.06)**	**0.024**	**0.12 (0.06)**	**0.027**
AD non-dementia group	Intercept	1.38 (0.20)	<0.001	1.35 (0.20)	<0.001	1.29 (0.18)	<0.001	1.52 (0.22)	<0.001	1.37 (0.19)	<0.001
	Age	0.00 (0.00)	0.275	0.00 (0.00)	0.295	0.00 (0.00)	0.418	0.00 (0.00)	0.189	0.00 (0.00)	0.250
	Female	0.03 (0.04)	0.467	0.06 (0.04)	0.100	0.03 (0.03)	0.327	0.03 (0.04)	0.424	0.04 (0.03)	0.236
	APOE ε4	0.00 (0.03)	0.940	-0.01 (0.03)	0.815	-0.04 (0.03)	0.231	0.01 (0.04)	0.884	-0.01 (0.03)	0.723
	CPV	0.17 (0.08)	0.028	0.15 (0.07)	0.043	0.13 (0.07)	0.058	0.18 (0.08)	0.034	**0.16 (0.07)**	**0.029**
AD dementia group	Intercept	1.02 (0.30)	0.001	1.27 (0.30)	<0.001	1.08 (0.29)	<0.001	1.27 (0.30)	<0.001	1.14 (0.28)	<0.001
	Age	0.00 (0.00)	0.453	0.00 (0.00)	0.934	0.00 (0.00)	0.534	0.00 (0.00)	0.804	0.00 (0.00)	0.645
	Female	0.07 (0.05)	0.180	0.07 (0.05)	0.170	0.08 (0.05)	0.086	0.06 (0.05)	0.236	0.07 (0.05)	0.146
	APOE ε4	0.02 (0.04)	0.707	0.02 (0.04)	0.652	-0.02 (0.04)	0.695	0.04 (0.05)	0.384	0.01 (0.04)	0.761
	CPV	0.12 (0.09)	0.214	0.07 (0.09)	0.428	0.04 (0.09)	0.695	0.10 (0.09)	0.291	0.09 (0.09)	0.331

Multivariate linear regression models were used to investigate the association between CPV and amyloid burden after adjusting for age at MRI scan, sex, and APOE ε4 carrier status.
*FDR correction for multiple comparison of four regional SUVRs
Bold indicates *P* or FDR-corrected *P* < 0.05.

Supplementary Table 3. Multivariate linear regression analyses for the effects of CPV on cognitive function in subgroup.

	Attention*		Language*		Visuospatial*		Memory*		Frontal/executive*		CDR-SOB	
	β (SE)	*P*	*β (SE)*	*P*	*β (SE)*	*P*	*β (SE)*	*P*	*β (SE)*	*P*	*β (SE)*	*P*
AD group	-0.27 (0.24)	0.261	0.31 (0.43)	0.466	-0.04 (0.63)	0.946	0.11 (0.24)	0.633	-0.25 (0.21)	0.242	0.69 (0.51)	0.176
AD non-dementia group	-0.20 (0.32)	0.543	0.43 (0.56)	0.441	0.38 (0.72)	0.604	0.34 (0.32)	0.288	-0.09 (0.25)	0.732	0.70 (0.42)	0.096
AD dementia group	-0.29 (0.37)	0.434	0.43 (0.65)	0.507	-0.46 (1.20)	0.703	-0.28 (0.36)	0.435	-0.43 (0.37)	0.252	0.99 (1.14)	0.385

Multivariate linear regression models were used to investigate the association between CPV and cognitive function after adjusting for sex. In terms of the model for CDR-SOB, age at MRI scan and years of education were additionally adjusted.
*FDR correction for multiple comparison of five cognitive domain
CDR-SOB = Clinical Dementia Rating Scale-Sum of boxes; SE = standard error;

SUPPLEMENTARY DATA

Supplementary Table 4. Comparison between whole patients on the AD continuum and subsample of longitudinal analysis.

	Whole patients on the AD continuum (n = 203)	Subsample of longitudinal analysis (n = 147)	*P*
**Demographic characteristics**			
Age at MRI scan	75.46 ± 7.06	75.12 ± 6.80	0.653
Female, n (%)	126 (62.1%)	89 (60.5%)	0.859
Education, y	10.74 ± 5.23	11.11 ± 5.15	0.515
MMSE	23.04 ± 3.91	23.67 ± 3.57	0.126
APOE ε4 carrier	106 (52.2%)	766 (51.7%)	>0.999
Vascular risk factors			
Hypertension	107 (52.7%)	75 (51.0%)	0.839
Diabetes mellitus	44 (21.7%)	28 (19.1%)	0.641
Dyslipidemia	59 (29.1%)	38 (25.9%)	0.588
**Neuropsychological tests**			
Attention/working memory	-0.20 ± 0.87	-0.12 ± 0.90	0.417
Language	-0.92 ± 1.54	-0.87 ± 1.59	0.760
Visuospatial	-0.87 ± 2.25	-0.70 ± 1.80	0.449
Memory	-1.28 ± 0.86	-1.21 ± 0.86	0.481
Frontal/executive	-0.78 ± 0.80	-0.76 ± 0.79	0.761
CDR-SOB	2.61 ± 2.05	2.46 ± 1.95	0.490
**Volumetric MRI measures**			
CPV, ratio of ICV × 10^3	1.50 ± 0.26	1.50 ± 0.26	0.927
LVV, ratio of ICV × 10^3	26.91 ± 10.22	26.17 ± 9.36	0.491
hippocampal volume, ratio of ICV × 10^3	4.96 ± 0.67	4.98 ± 0.68	0.865
WMHV, ratio of ICV × 10^3	3.29 ± 3.20	3.00 ± 2.96	0.392
ICV, mL	1322.01 ± 125.19	1329.80 ± 130.09	0.572
**^{18}F-FBB PET scan**			
Frontal SUVRs	1.48 ± 0.19	1.46 ± 0.18	0.329
Parietal SUVRs	1.49 ± 0.19	1.47 ± 0.18	0.360
Lateral temporal SUVRs	1.39 ± 0.18	1.38 ± 0.17	0.530
Anterior/posterior cingulate SUVRs	1.57 ± 0.20	1.56 ± 0.19	0.408
Global SUVRs	1.47 ± 0.18	1.45 ± 0.17	0.347

Values are expressed as mean ± standard deviation or number (percentage).
P values are the results of independent *t* test, chi-square tests or Fisher's Exact tests, as appropriate.

© 2024. Jeong SH et al. Published online at http://www.aginganddisease.org/EN/10.14336/AD.2024.0118

SUPPLEMENTARY DATA

Supplementary Table 5. Demographic characteristics of study participants who were included in longitudinal analysis.

	AD non-dementia (n = 94)	AD dementia (n = 53)	*P*
**Demographic characteristics**			
Age at MRI scan	74.85 ± 6.89	75.59 ± 6.68	0.532
Female, n (%)	53 (56.4%)	36 (67.9%)	0.231
Education, y	11.96 ± 4.87	9.58 ± 5.32	0.007
MMSE	24.90 ± 2.67	21.47 ± 3.93	<0.001
APOE ε4 carrier	50 (53.2%)	26 (49.1%)	0.757
Vascular risk factors			
Hypertension	49 (52.1%)	26 (49.1%)	0.853
Diabetes mellitus	20 (21.3%)	8 (15.1%)	0.485
Dyslipidemia	24 (25.5%)	14 (26.4%)	>0.999
**Neuropsychological tests**			
Attention/working memory	-0.00 ± 0.92	-0.34 ± 0.82	0.030
Language	-0.67 ± 1.61	-1.23 ± 1.49	0.042
Visuospatial	-0.47 ± 1.52	-1.12 ± 2.18	0.065
Memory	-1.07 ± 0.87	-1.48 ± 0.76	0.006
Frontal/executive	-0.57 ± 0.72	-1.09 ± 0.80	<0.001
CDR-SOB	1.60 ± 1.03	4.04 ± 2.25	<0.001
**Volumetric MRI measures**			
CPV, ratio of ICV × 10^3	1.49 ± 0.27	1.50 ± 0.26	0.816
LVV, ratio of ICV × 10^3	25.44 ± 9.33	27.46 ± 9.36	0.211
hippocampal volume, ratio of ICV × 10^3	5.00 ± 0.67	4.93 ± 0.70	0.551
WMHV, ratio of ICV × 10^3	3.04 ± 3.00	2.92 ± 2.91	0.815
ICV, mL	1360.58 ± 122.57	1275.21 ± 126.09	<0.001
**^{18}F-FBB PET scan**			
Frontal SUVRs	1.43 ± 0.18	1.52 ± 0.17	0.004
Parietal SUVRs	1.44 ± 0.18	1.52 ± 0.17	0.007
Lateral temporal SUVRs	1.35 ± 0.15	1.44 ± 0.18	0.004
Anterior/posterior cingulate SUVRs	1.53 ± 0.19	1.60 ± 0.17	0.017
Global SUVRs	1.42 ± 0.16	1.51 ± 0.17	0.004

Values are expressed as mean ± standard deviation or number (percentage).
P values are the results of independent *t* test, chi-square tests or Fisher's Exact tests, as appropriate.

© 2024. Jeong SH et al. Published online at http://www.aginganddisease.org/EN/10.14336/AD.2024.0118
